# Recovery from Backward Curvature of the Spine, with Removal of the Deformity

**Published:** 1867-06

**Authors:** Julien S. Sherman

**Affiliations:** Chicago


ARTICLE XXIV.

### RECOVERY FROM BACKWARD CURVATURE OF THE SPINE—WITH REMOVAL OF THE DEFORMITY.

By JULIEN S. SHERMAN, M.D., Chicago.

W. H. B., of Chicago, *æt.* 25 years, applied for treatment in Oct. 1866, stating that a year previous he had fallen from a window, injuring his back to such an extent as to confine him to his bed for several months. He recovered sufficiently to be able to partially attend to his business, but on any unusual exertion, pain would return at the seat of injury and oblige him to desist from his labor. There was slight numbness of the left leg and thigh; pressure upon the shoulders caused pain at the injured spot.

On examination of the back, the spinous process of the last dorsal vertebra showed a decided projection, with all the characteristics of "Potts' disease." This case was also, at the same time, examined by Dr. ANDREWS.

An apparatus, to relieve pressure and separate the bodies of the diseased vertebræ, was applied and worn constantly, day and night. Great relief from suffering was immediately experienced, and at present (May 9, '67) the deformity is scarcely perceptible, and the locality of the disease can be discovered only by a very careful examination. The back is perfectly strong, and no evidence of disease remains. The patient will be required to wear the apparatus a short time longer, when it will be discarded.

The arrest of the deformity and cure, by anchylosis, of the vertebræ in their mal-position, is the general result of treatment in these cases. The disease being essentially one of inflammation of the bone and soft structures of the spinal column, followed by absorption and, frequently, caries, it is greatly aggravated by pressure.

The present case illustrates the fact, that when the weight of the body is effectually and for a sufficient length of time removed, the inflammation will subside and new bone frequently be deposited to take the place of that destroyed, and the normal symmetry of the column be restored. This favorable result cannot be obtained in all cases. To insure such a recovery, there should be no anchylosis in any part of the diseased surfaces, as this would prevent a sufficient separation of the bodies to restore the proper axis of the spine. The case should not be advanced to suppuration or formation of fistulæ, for such a condition would render the application of a proper apparatus difficult. A satisfactory result is much more likely to follow when the inflammation is of a simple character and not complicated with, or dependent on, tubercle.

